# Genome Editing of Rice *eIF4G* Loci Confers Partial Resistance to Rice Black-Streaked Dwarf Virus

**DOI:** 10.3390/v13102100

**Published:** 2021-10-18

**Authors:** Wei Wang, Shuhui Ma, Peng Hu, Yinghua Ji, Feng Sun

**Affiliations:** 1 Jiangsu Key Laboratory for Food Quality and Safety-State Key Laboratory Cultivation Base, Institute of Plant Protection, Jiangsu Academy of Agricultural Sciences, Nanjing 210014, China; 18256668028@163.com (W.W.); m1195857235@163.com (S.M.); 15279889632@163.com (P.H.); 2 College of Plant Protection, Nanjing Agricultural University, Nanjing 210095, China; 3 The State Key Laboratory of Crop Genetics and Germplasm Enhancement, Nanjing Agricultural University, Nanjing 210095, China

**Keywords:** RBSDV, *eIF4G*, genome editing, CRISPR/Cas9, virus resistance

## Abstract

Rice black-streaked dwarf disease, caused by rice black-streaked dwarf virus (RBSDV), is a serious constraint in Chinese rice production. Breeding disease-resistant varieties through multigene aggregation is considered an effective way to control diseases, but few disease-resistant resources have been characterized thus far. To develop novel resources for resistance to RBSDV through CRISPR/Cas9-mediated genome editing, a guide RNA sequence targeting exon 1 of *eIF4G* was designed and cloned into a binary vector, pHUE401. This recombinant vector was used to generate mutations in the rice cultivar Nipponbare via *Agrobacterium*-mediated transformation. This approach produced heritable homozygous mutations in the transgene-free T1 generation. Sequence analysis of the *eIF4G* target region from T1 transgenic plants identified 3 bp deletion mutants, and analysis of the predicted amino acid sequence identified one amino acid deletion in mutants that possess near full-length eIF4G. Furthermore, our data suggest that *eIF4G* may plays an important role in rice normal development, as there were no *eIF4G* knock-out homozygous mutants in T1 generation plants. When homozygous mutant lines were inoculated with RBSDV, they exhibited enhanced tolerance to virus infection, without visibly affecting plant growth and development. However, the *eif4g* mutant plants showed the same sensitivity to rice stripe virus (RSV) infection as wild-type plants. Notably, the wild-type and mutant N-termini of eIF4G interacted directly with RBSDV P8 in yeast and in planta. Additionally, compared to wild-type plants, the *eIF4G* transcript level was reduced twofold in the mutant plants. These results indicate that site-specific mutation of rice *eIF4G* successfully conferred partial resistance specific to RBSDV associated with less transcription of *eIF4G* in mutants. Therefore, this study demonstrates that the novel *eIF4G* alleles generated by CRISPR/Cas9 represent valuable disease-resistant resources that can be used to develop RBSDV-resistant varieties.

## 1. Introduction

Rice black-streaked dwarf virus (RBSDV), transmitted by the small brown planthopper (SBPH: *Laodelphax striatellus* Fallén), mainly causes rice black-streaked dwarf disease and maize rough dwarf diseases, which lead to serious cereal crop yield losses in China [1,2]. Rice plants infected by RBSDV typically exhibit acute growth abnormalities, such as severe dwarfism, dark green and stiff leaves, and tumors on leaves and stems [3,4,5]. Diseased rice plants generally reduce the yield by 10–40%, and severely diseased rice plants even have no grains [6].

RBSDV is a double-stranded RNA virus and belongs to the genus Fijivirus within the family Reoviridae [7]. RBSDV virus particles are icosahedral, with double-layer shells containing spikes, and contain 10 double-stranded RNAs, S1–S10 [8,9]. RBSDV genomic segments encode six structural proteins P1, P2, P3, P4, P8 and P10 in its viral particle and seven nonstructural proteins P5-1, P5-2, P6, P7-1, P7-2, P9-1 and P9-2 [10,11]. P8, the minor core protein of the RBSDV virion, can enter the nucleus of the plant and insect cells and possesses transcriptional repression activity in tobacco suspension cells [12]. SP8 encoded by SRBSDV, a closely related Fijivirus, can interact with rice auxin response factor 17 (OsARF17) to facilitate virus infection by impairing auxin-mediated antiviral defense [13]. In addition, a recent study showed that RBSDV P8 interacts with maize AKINß protein (ß subunit of *Arabidopsis* SNF1 kinase homolog in maize) and facilitates viral accumulation in maize [14]. All these results suggest that P8 plays an important role during the process of virus-host interaction.

To manage rice black-streaked dwarf disease caused by RBSDV, insecticides are frequently employed to control SBPH vectors, accompanied by increasing environmental and economic concerns [15]. Recently, genetically modified crops resistant to viruses have been shown to be an economically and environmentally feasible alternative [16,17]. Among these antiviral strategies, artificial deletion and point mutations using the clustered regularly interspersed palindromic repeats (CRISPR)/CRISPR-associated protein 9 (CRISPR/Cas9) technique in the gene encoding host factors that are essential for the virus life cycle provide virus resistance without hampering the overall health of the plant [18,19]. Eukaryotic translation initiation factor (eIF) genes, such as eukaryotic translation initiation factor 4E (*eIF4E*) and eukaryotic translation initiation factor 4G (*eIF4G*), and their isoforms are required for RNA viruses to translate viral RNAs [20,21]. According to this knowledge, using CRISPR/Cas9 technology, sequence-specific point mutations at *eIF(iso)4E* confer complete resistance to turnip mosaic virus (TuMV) in *Arabidopsis* plants [22]. Similarly, the use of CRISPR/Cas9 to knock out the *eIF4E* gene in cucumbers resulted in resistance to cucumber vein yellowing virus (CYVY), zucchini yellow mosaic virus (ZYMV) and papaya ring spot mosaic virus-W (PRSV-W) under greenhouse conditions [23]. In rice plants, novel *eIF4G* alleles produced through the CRISPR/Cas9 system confer resistance to rice tungro spherical virus (RTSV) [24]. Thus, the novel *eIF4E*/*eIF4G* alleles generated by CRISPR/Cas9-mediated genome editing are expected to be used in the development of virus-resistant crop plants.

In this study, to introduce novel alleles of rice *eIF4G* loci, we mutated *eIF4G* in *Oryza sativa var. japonica cv.* Nipponbare using CRISPR/Cas9-mediated genome editing technology and generated the T0 transgenic rice plant that carried *eIF4G* mutants. The selected T2 rice plants that carried an edited *eIF4G* gene but not the introduced transgene were tested for resistance to RBSDV. Compared with wild-type plants, homozygous rice plants with edited *eIF4G* genes had significantly increased tolerance to RBSDV, without affecting rice stripe virus (RSV) infection and normal plant growth. Furthermore, the wild-type and mutant N-termini of eIF4G interacted directly with RBSDV P8 in yeast and in planta. Altogether, this study provides important information on the mechanism of interaction between RBSDV and rice plants, and novel *eIF4G* alleles represent valuable materials for the development of RBSDV-resistant varieties.

## 2. Materials and Methods

### 2.1. Sources of Virus, Vectors, and Plant Materials

RBSDV or RSV-infected rice plants were obtained from fields in Jiangsu Province, China, and the virus was confirmed by RT-PCR assay [4]. The nonviruliferous small brown planthopper (SBPH) strains were collected from fields in Jiangsu Province, China, and reared on rice seedlings (*Oryza sativa* L. cv. Wuyujing No. 3) in glass incubators in an insect-rearing room at 25 °C. The rice cultivar Nipponbare (*O. sativa* L. subsp. japonica, cv. NIP) was used as a background to generate *eIF4G* CRISPR/Cas9 mutant (*eif4g*) lines. All rice plants were grown in the greenhouse at 28–30 °C with a 12 h light/12 h dark photoperiod.

### 2.2. Viruses Inoculation Assay

Nonviruliferous instar nymphs of SBPHs were fed on RBSDV(or RSV)-infected rice plants for 7 days to acquire the virus. Then, the nymphs were transferred to healthy rice seedlings and allowed to feed for 10 days. The percentage of viruliferous SBPHs was determined by dot enzyme-linked immunosorbent assay (Dot-ELISA) using the monoclonal antiRBSDV or antiRSV antibody [25]. Transgenic and wild-type rice plants at the 2 two-leaf-stage were inoculated with 2 viruliferous SBPHs per plant and were kept in a glass incubator containing 30 plants. SBPHs were moved after a 2-day inoculation access period. The inoculated seedlings were transplanted into an experimental field at the Jiangsu Academy of Agricultural Sciences for symptom development, and nonviruliferous insects were used for mock inoculation.

### 2.3. Evaluation of RBSDV or RSV Resistance

The RBSDV or RSV inoculated rice seedlings were transplanted into an experimental plot at the Jiangsu Academy of Agricultural Sciences, Nanjing, China. The seedlings were grown under a standard agricultural practice without the using of pesticides during growth. Four weeks after transplanting (28 dpi), the RBSDV or RSV incidence was recorded for the wild-type and its mutant lines, respectively. Three replicates were used for each treatment, and each replicate contained at least 30 rice seedlings, and the disease incidence of each rice line (%) was determined use the following formula: number of RBSDV (or RSV)-infected plants/total number of plants counted × 100. In addition, 10 rice seedlings were pooled at 28 dpi for qRT-PCR and Western blot analysis.

### 2.4. DNA Constructs and Transgenic Plants

The N-terminus of wild-type eIF4G (eIF4G-N) or mutant eIF4G (eIF4G-N^mu^) and full-length RBSDV P8 sequences were amplified by PCR from RBSDV-infected rice plants, and the PCR products were cloned individually into pDONR-zero vectors using BP Clonase Enzyme Mix (Invitrogen, Carlsbad, CA, USA). The eIF4G-N or eIF4G-N^mu^ and P8 sequences were then transferred to the appropriate destination vectors using LR Clonase (Invitrogen) for yeast two-hybrid and luciferase complementary imaging assays.

For gene editing, rice eIF4G-specific sgRNA was designed by the CRISPR PLANT website (http://www.genome.arizona.edu/crispr/CRISPRsearch.html, accessed on 6 October 2018). These sgRNA oligos were synthesized (Genscript, Nanjing, China) and cloned into the pHUE401 vector by Golden Gate cloning, as previously described [26]. CRISPR/Cas9 vectors were introduced into *Agrobacterium tumefaciens* strain GV3101 by electroporation and transformed into Nipponbare by Agrobacterium-mediated transformation. The primers used in this study are listed in Supplementary Table S1.

### 2.5. Western Blot Assay

Western blotting was performed according to the protocol described previously [27]. Briefly, rice total proteins were extracted in 2× SDS-loading buffer (100 mM Tris-HCl (pH 6.8), 4% SDS, 0.2% bromophenol blue, 20% glycerol, 2% beta-mercaptoethanol), separated by 10% SDS-PAGE and transferred onto PVDF membranes. The membranes were blocked and inoculated with an antiRBSDV P6 or antiRSV SP (provided by Jianxiang Wu, Zhejiang University, Hangzhou, China) or antiactin (Enogene, Nanjing, China) or antiRubisco (Sangon Biotech, Shanghai, China) antibody for 2 h at room temperature. An HRP-conjugated antirabbit antibody was used as the secondary antibody (Abmart, Shanghai, China). Signals were developed in ECL buffer (Vazyme Biotech, Nanjing, China) and recorded with a Tanon 5200 Luminescent Imaging Workstation (Tanon, Shanghai, China).

### 2.6. Quantitative Reverse-Transcription PCR (qRT-PCR)

Total RNA was extracted from 100 mg rice plants with RNAiso Plus reagent (Takara, Dalian, China) according to the manufacturer’s instructions. cDNA was synthesized with the iScript™ cDNA Synthesis Kit (Bio-Rad, Hercules, CA, USA) using random hexamers as primers. qPCR was performed using SsoFast EvaGreen Supermix (Bio-Rad, Hercules, CA, USA) on a Bio-Rad iQ5 qRT-PCR system with gene-specific primers (Supplementary Table S1). The results were normalized to reference gene expression (*UBQ10*) using the 2^−ΔΔCt^ method reported previously [28].

### 2.7. Yeast Two-Hybrid Assay

The Matchmaker Gold Y2H system (Clontech, Mountain View, CA, USA) was used to identify interactions between eIF4G-N (or eIF4G-N^mu^) and RBSDV P8. The CDSs of eIF4G-N (1–1053) (or eIF4G-N^mu^) and RBSDV P8 were cloned into pGBK-DC and pGAD-DC gateway destination vectors (provided by Xiuren Zhang, Texas A&M University, College Station, USA), respectively, by the LR reaction (Invitrogen, Carlsbad, CA, USA). The plasmids pGBK-eIF4G-N (or pGBK-eIF4G-N^mu^) and pGAD-P8 were cotransformed into the Y2H Gold (Clontech) yeast strain and selected on medium lacking leucine, tryptophan, histidine and adenine. The plasmids pGBK-53 and pGAD-T7 were cotransformed into the Y2H Gold yeast strain as a positive control.

### 2.8. Luciferase Complementary Imaging Assay (LCI Assay)

The CDSs of eIF4G-N (1–1053) (or eIF4G-N^mu^) and RBSDV P8 were cloned into pCAMBIA-DC-NLuc and pCAMBIA-CLuc-DC (provided by Xiuren Zhang, Texas A&M University, College Station, USA), respectively, by the LR reaction. Then, the constructs were transformed into *A. tumefaciens* strain GV3101. Agrobacteria harboring the pCAMBIA-eIF4G-N-Nluc and pCAMBIA-CLuc-P8 plasmids co-infiltrated into 4-week-old *N. benthamiana* leaves. After 2 days, the infiltrated leaves were sprayed with 10 mM luciferin (Promega, Madison, WI, USA) and photographed using the Tanon 5200 Luminescent Imaging Workstation (Tanon, Shanghai, China).

## 3. Results

### 3.1. Generation and Characterization of Genome-Edited eIF4G Rice Plants

**Figure 1:**
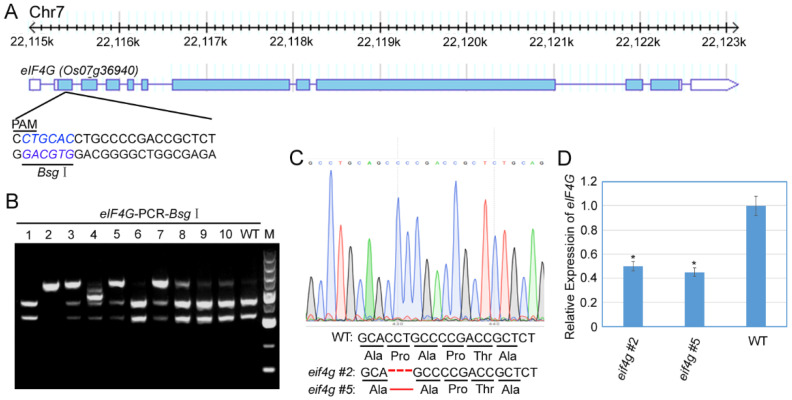
Genomic editing of rice *eIF4G* by CRISPR/Cas9. (**A**) Schematic diagram of the CRISPR/Cas9 target sites in *eIF4G*. The sgRNA target sequences are shown in black letters, and *Bsg* I digestion sequences are shown in blue letters. (**B**) Mutation analysis of 10 T0 transgenic lines by *Bsg* I digestion of PCR fragments. (**C**) DNA and amino acid sequence analysis of mutated alleles identified from cloned PCR fragments of two representative T1 transgenic lines. DNA and amino acid deletions are denoted by red dashes. (**D**) qRT-PCR analysis of *eIF4G* mRNA transcription levels in mutants and WT rice plants. Signal intensities for each transcript were normalized to those for UBQ. All data are means ± SD (*n* = 3).The statistical significance of the difference between the *eif4g* mutant lines and wild-type plants was determined using Student’s *t*-test. * *p* < 0.05.

To investigate the function of rice *eIF4G* loci in RBSDV infection, we aimed to use CRISPR/Cas9-mediated targeted genome editing of *eIF4G* (LOC_Os07g36940) to develop RBSDV-resistant rice plants. To design sgRNAs, we identified *eIF4G* target sgRNA sequences using CRISPR PLANT tools (http://www.genome.arizona.edu/crispr/CRISPRsearch.html, accessed on 6 October 2018). sgRNAs targeting the first exon with the *Bsg* I restriction site were used for genome editing (Figure 1A). We cloned this sgRNA into a binary vector (pHUE401) [26], and this pHUE401-eIF4G CRISPR/Cas9 construct was introduced into rice plants (Nipponbare) by Agrobacterium-mediated transformation.

Ten rice plants were generated by hygromycin selection and analyzed for CRISPR/Cas9-induced mutation in *eIF4G* by restriction enzyme digestion of a PCR fragment encompassing the target region (Figure 1B). These results showed that 50% of the T0 transgenic lines (#2, #3, #4, #5, #7) had a mutation in the target sites, Line #2 had biallelic mutations, and the others had monoallelic mutations. We cloned and sequenced the PCR fragments from T1 generation plants of two lines (#2 and #5) and found that three nucleotides were deleted from the target sequences, as shown in Figure 1C. Analysis of the translated protein sequence identified that 79-site proline were deleted in the eIF4G protein in the two mutant lines (#2 and #5) (Figure 1C). To determine the effect of mutants on the *eIF4G* expression level, we analyzed the *eIF4G* mRNA transcription level by qRT-PCR assay using homozygous mutant lines (#2 and #5). The results showed that, compared with wild-type (WT) plants (Nipponbare), the expression of *eIF4G* RNA transcript was significantly reduced in the mutant plants *eif4g* #2 and *eif4g* #5 (Figure 1D).

### 3.2. Segregation of Mutation and cas9 from the Transgene in the T1 Generation

**Figure 2:**
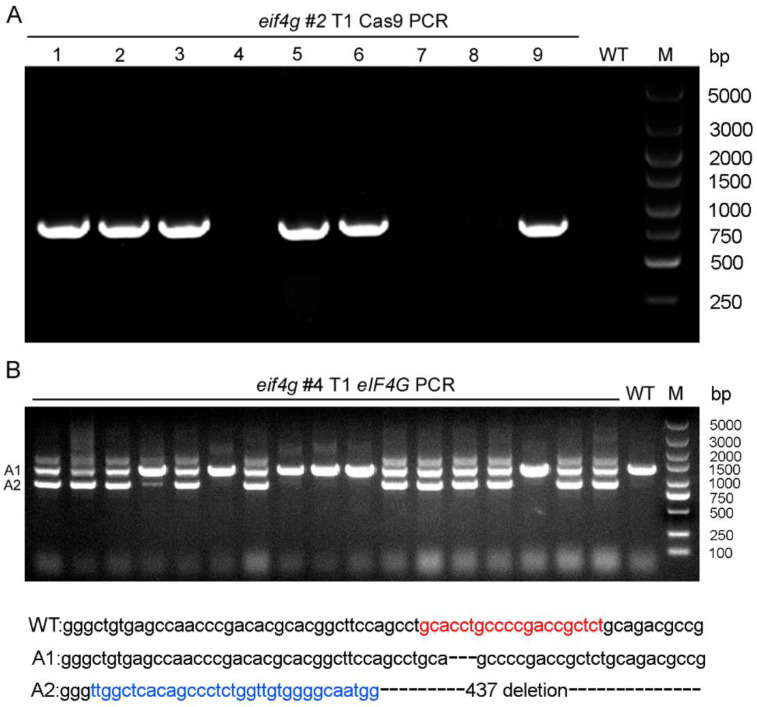
Transgene cas9 and mutation screening in T1 generation rice plants. (**A**) Transgene screening of *eIF4G*-targeted T1 transgenic plants by genomic DNA PCR. WT: wild-type plants. M: DNA marker. (**B**) *eif4g* mutant screening in *eIF4G*-targeted T1 transgenic plants (#4) by genomic DNA PCR. WT: wild-type plants; DNA sequence deletions are denoted by black dashes, and DNA sequence insertions are denoted by blue letters. The sgRNA target sequences are shown in red letters. M: DNA marker.

Among the T0 generation of *eif4g* mutants, we selected the #2, #4 and #5 plants from which to generate T1 lines. First, we tested the presence of transgenes by PCR with Cas9-specific primers within T1 plants from *eif4g* #2, which was a biallelic mutant in the T0 generation. Among 9 plants derived from *eif4g* #2, 6 carried Cas9 transgenes (1, 2, 3, 5, 6, 9), and 3 did not (4, 7, 8) (Figure 2A). Sequencing of amplicons of the target region showed that all T1 generations of *eif4g* #2 were homozygous mutants. These results indicated that the transgene-free T1 generation of *eif4g* #2 plants (4, 7, 8) were all homozygous mutants.

For the T0 generation of *eif4g* #4 mutants containing *eIF4G* small fragments (A2), we analyzed these *eIF4G* small fragments in the T1 population plants with PCR. Our results showed that there were no *eIF4G* small fragment homozygous mutants in the T1 generation of *eif4g* #4 (Figure 2B). DNA sequencing of *eIF4G* small fragments showed that this mutant carried a 33 bp insertion and 437 bp deletion (Figure 2B). These results indicate that *eIF4G* plays an important role in rice plant development and that knocking out *eIF4G* may be lethal.

### 3.3. eIF4G Edited Rice Plants Confer Partial Resistance to RBSDV

**Figure 3:**
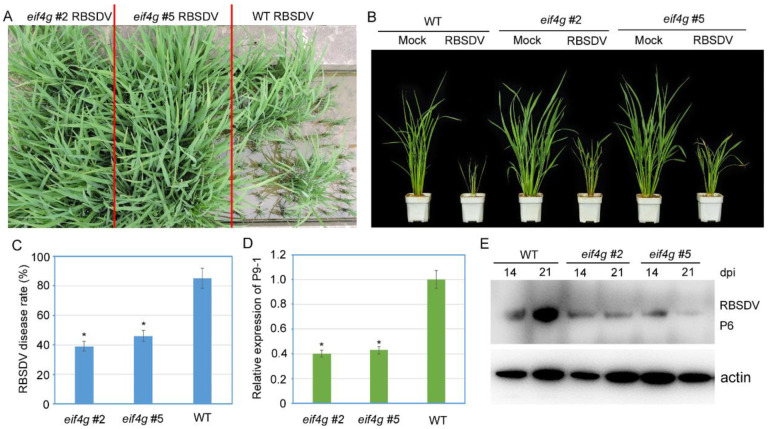
Evaluation of rice black-streaked dwarf virus (RBSDV) resistance of rice *eif4g* mutant lines. (**A**,**B**) Disease symptoms of mock-inoculated and RBSDV-infected *eif4g* mutant lines (*eif4g* #2, *eif4g* #5) and wild-type (WT, Nipponbare) rice plants. Photographs were taken at 28 days postinoculation (dpi). (**C**) Incidence of RBSDV disease rate in *eif4g* mutant lines (*eif4g* #2, *eif4g* #5) and wild-type (WT, Nipponbare) rice plants at 28 dpi. (**D**) qRT-PCR analysis of RBSDV *P9-1* mRNA transcription levels in *eif4g* mutant lines (*eif4g* #2, *eif4g* #5) and WT rice plants at 28 dpi. Signal intensities for each transcript were normalized to the signal intensity for UBQ. (**E**) Western blot analysis of RBSDV-encoded P6 protein accumulation in virus-infected *eif4g* mutant lines (*eif4g* #2, *eif4g* #5) and wild-type (WT, Nipponbare) rice plants using a P6-specific antibody. The actin protein level served as a loading control. Rice plants were all collected at 28 dpi. All data are means ± SD (*n* = 3).The statistical significance of the difference between the *eif4g* mutant lines and wild-type plants was determined using Student’s *t*-test. * *p* < 0.05.

To examine whether these *eif4g* mutant rice plants edited by CRISPR/Cas9 affect RBSDV resistance, two homozygous T2 populations (*eif4g* #2 and *eif4g* #5) and wild-type plants were inoculated with RBSDV. The wild-type plants showed typical RBSDV symptoms at 21 days postinoculation (DPI), including severe dwarfing and dark green leaves. Both *eif4g* mutant rice lines showed mild symptoms (Figure 3A,B). At 28 dpi, the RBSDV disease incidence of *eif4g* mutant lines (*eif4g* #2 and *eif4g* #5) was significantly lower than the RBSDV disease incidence of the wild-type plants (Figure 3C). To confirm RBSDV virus infection, we analyzed viral RNA accumulation by qRT-PCR using RBSDV P9-1 gene-specific primers and virus P6 protein accumulation by Western blot assay. The results showed that compared with wild-type plants, the accumulation levels of RBSDV P9-1 RNA in the *eif4g* mutant lines were significantly decreased (Figure 3D). Similarly, the *eif4g* mutant lines accumulated less P6 protein than wild-type plants at 21 dpi (Figure 3E). These results all showed that the *eIF4G*-edited lines conferred partial resistance to RBSDV.

To determine if the *eif4g* mutant rice plants have a broader spectrum of resistance to virus infection, these mutant plants were inoculated with rice stripe virus (RSV), which is type species in the genus *Tenuivirus* and transmitted by small brown planthopper (SBPH). The RSV infection assay showed that the *eif4g* mutant rice plants developed severe stunting symptom similar to wild-type plants (Figure S1A). At 28 dpi, the RSV disease incidence of *eif4g* mutant lines (*eif4g* #2 and *eif4g* #5) was also similar to wild-type plants (Figure S1B). qRT-PCR and Western blot results both showed that RSV SP mRNA and proteins accumulated in *eif4g* mutant plants were comparable with wild-type plants (Figure S1C,D). These data indicated that the *eif4g* mutant plants had no effect on RSV tolerance.

**Figure 4:**
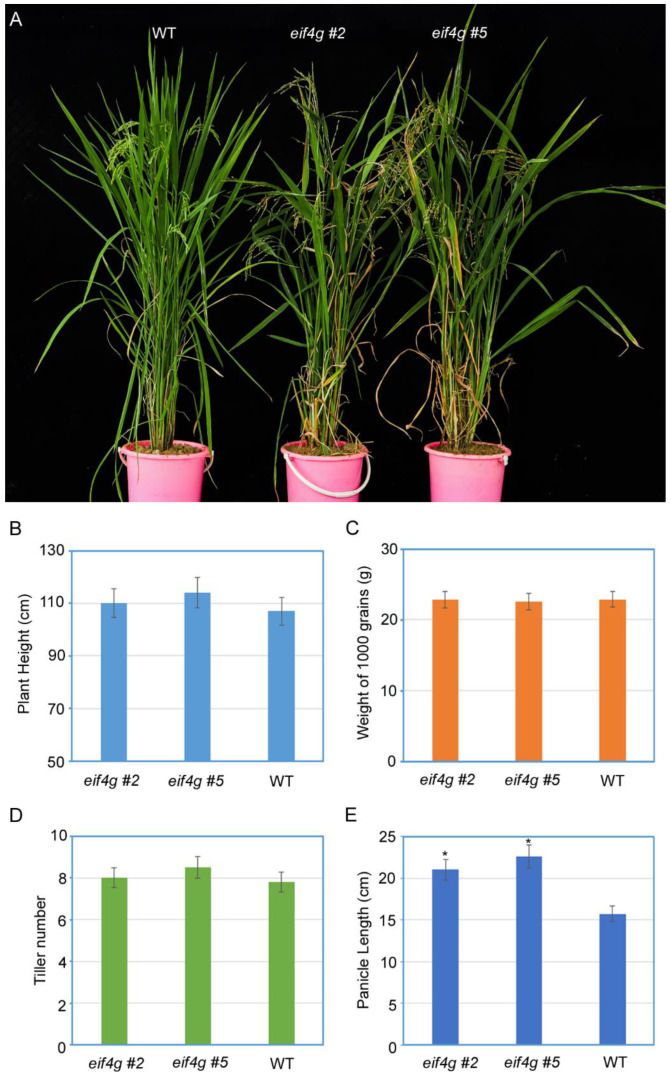
Examination of the agronomic parameters of rice *eif4g* mutant lines. (**A**) Development phenotype of *eif4g* mutant lines (*eif4g* #2, *eif4g* #5) and wild-type (WT, Nipponbare) rice plants. Photographs were taken at 60 days after germination. (**B**–**E**) The agronomic parameters of *eif4g* mutant lines (*eif4g* #2, *eif4g* #5) and wild-type (WT, Nipponbare) rice plants. All data are means ± SD (*n* = 20).The statistical significance of the difference between the *eif4g* mutant lines and wild-type plants was determined using Student’s *t*-test. * *p* < 0.05.

To clarify the effect of *eif4g* mutants on plant growth and development, agronomic parameters such as plant height, weight of 1000 grains, tiller number per plant and panicle length were measured in two *eif4g* mutant lines and wild-type plants. Compared to wild-type plants, *eif4g* mutants exhibited similar agronomic parameters but a significant increase in panicle length (Figure 4).

### 3.4. The N-Terminus of eIF4G Directly Interacted with RBSDV P8 in Yeast and Plants

**Figure 5:**
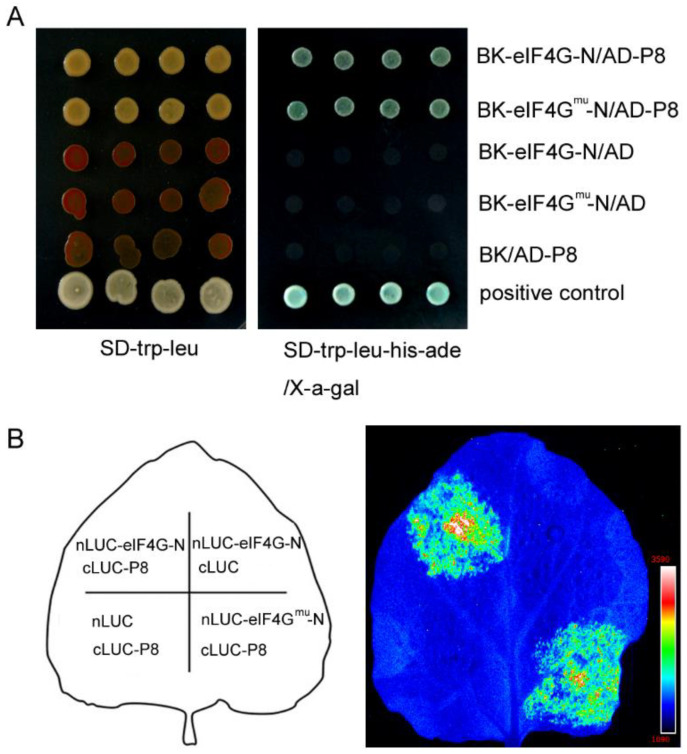
N-terminus of eIF4G (eIF4G-N) interacts with RBSDV P8 in yeast and in planta. (**A**) Yeast two-hybrid assays show that eIF4G-N interacts with the RBSDV P8 protein. The plasmid combinations indicated on the right side of the image panel were cotransformed into yeast strain Y2H Gold, and the yeast cells were spotted on SD/-Trp/-Leu and SD/-Trp/-Leu/-His/-Ade selective medium with X-a-gal and grown at 30 °C for 3–4 d. Positive control, full-length pGADT7–53 and pGBKT7-T7. (**B**) The luciferase complementation imaging assay indicates that eIF4G-N interacts with the RBSDV P8 protein in planta. The plasmid combinations indicated on the left side of the image panel were coexpression *Nicotiana benthamiana* leaves by Agrobacterium-mediated infiltration. After 48 h, *N. benthamiana* leaves were sprayed with Lucifer, and a fluorescence signal appeared where eIF4G-N-nLuc or eIF4G^mu^-N-nLuc was coexpressed with cLuc-P8. eIF4G^mu^, eIF4G mutant protein generated by CRISPR/Cas9.

To understand the molecular mechanism of eIF4G-mediated RBSDV resistance, we used a yeast two-hybrid assay to screen nine RBSDV proteins (P5-1, P5-2, P6, P7-1, P7-2, P8, P9-1, P9-2 and P10) for eIF4G interactions using the N-terminus of eIF4G (eIF4G-N) fused to the GAL4 DNA binding domain (BK-eIF4G-N) as bait. The yeast two-hybrid results indicated that cotransformation with BK-eIF4G-N and AD-P8 vectors allowed yeast to grow on selective media without histidine and adenine, in contrast to the negative controls (Figure 5A). The interaction was also detected by analyzing LacZ reporter gene activation. This result indicates a direct interaction between eIF4G-N and P8. To evaluate this interaction in planta, luciferase complementary imaging (LCI) assays were performed. In LCI experiments, the N- and C-terminal parts of luciferase (nLuc and cLuc) were fused to eIF4G-N and P8, respectively, and were coexpressed in *Nicotiana benthamiana* leaves by Agrobacterium-mediated infiltration. In our LCI assay, coexpression of eIF4G-N-nLuc and P8-cLuc restored the luciferase catalytic activity, suggesting that P8 was physically close to the eIF4G protein in vivo (Figure 5B). To further examine whether P8 interacted with the eIF4G mutant protein (eIF4G^mu^) generated by CRISPR/Cas9, we conducted yeast two-hybrid and LCI assays. The results of Figure 5 show that the eIF4G mutant did not affect the interaction with the RBSDV P8 protein. Together, these assays clearly indicated that eIF4G-N and P8 interact in vivo.

## 4. Discussion

RBSDV causes rice black-streaked dwarf disease, which occurs in East Asian countries and is responsible for severe rice yield losses [29]. To manage rice RBSDV disease, in the absence of commercially resistant varieties, the application of insecticides against SPBH vectors is the most common approach. However, this approach has polluted the environment and led to the development of insecticide-resistant SBPHs [30]. Therefore, genetic resistance for disease control is the most sustainable and environmentally friendly control strategy [31]. CRISPR/Cas9 has become a powerful tool for developing resistance resources against virus infection in various plant species [32]. In our study, we developed CRISPR/Cas9-edited rice *eif4g* mutants through Agrobacterium-mediated transformation. Sequencing of the *eIF4G* target region revealed that indels occurred 3 bp upstream of the PAM site, creating a 3-bp deletion or 1 amino acid deletion (Figure 1C). Notably, we obtained two homozygous *eif4g* mutant lines in the T2 generation, which shared the same mutant type and were free of the Cas9 transgene (Figure 1C). These two *eif4g* mutant lines confer RBSDV partial resistance but without any growth and development defects (Figure 3 and Figure 4). Thus, in this study, the RBSDV-tolerance plants generated by CRISPR/Cas9 may represent a valuable resistance material and improve RBSDV resistance by molecular rice breeding.

Plant viruses, obligate parasites, recruit cellular translation factors to synthesize their proteins, regulate their replication and potentiate their local and systemic movement. Many natural plant virus recessive resistance genes have been targeted to translation factors eIF4E and eIF4G or their isoforms, eIFiso4E and eIFiso4G [33]. Editing or knockout of one of these factors confers virus resistance but does not interfere with the overall health of the plant. Previous studies have shown that different potyviruses selectively recruit eIF4G or its isoforms to accomplish their infectious cycles. In Arabidopsis plants, the eIF4G knockout mutant is resistant to clover yellow vein virus (ClYVV), the eIF(iso)4G1 mutant is resistant to plum pox virus (PPV) and lettuce mosaic virus (LMV), and the eIF(iso)4G1 eIF(iso)4G2 double mutant is resistant to turnip mosaic virus (TuMV) [34]. Similarly, Arabidopsis homozygous for T-DNA insertions in the eIF4G gene displays reduced multiplication of cucumber mosaic virus (CMV) and turnip crinkle virus (TCV) [35]. In rice plants, the natural resistance gene to rice yellow mottle virus (RYMV) is correlated with mutations in eIF(iso)4G [36], and novel eIF4G alleles generated using CRISPR/Cas9 result in resistance to rice tungro spherical virus (RTSV) [24]. Here, we show that new rice eIF4G alleles generated by CRISPR/Cas9 technology confer RBSDV partial resistance without hindering plant growth and affecting RSV infection (Figure S1). These data suggest that RBSDV and RSV recruit different translation initiation factors to complete virus life cycles. Nevertheless, the resistance level to RBSDV was not completely immune (Figure 3 and Figure 4), which can be explained because RBSDV may use eIF(iso)4G for virus replication and systemic movement and therefore can cause mild symptoms. RBSDV resistance genetic analysis also demonstrates that several quantitative trait loci (QTLs) have been identified in rice genetic populations [37,38]. These results suggest that RBSDV may recruit eIF4G and eIF(iso)4G to complete its disease cycle. Thus, eIF4G and eIF(iso)4G double-mutant plants would be expected to show more resistance to RBSDV.

Previous studies of potyviruses suggest that mutations in the eIF4E/(iso)4E protein interrupt the interaction between viral genome-linked protein (VPg) and eIF4E/(iso)4E and confer plant resistance to virus infection [39,40]. The VPg of potato virus A (PVA), as a cap-like structure, recruits host eIF(iso)4E via a specific motif for PVA RNA stabilization, as well as viral protein synthesis [41]. Similarly, rice yellow mottle virus (RYMV) VPg interacts with the central domain of rice eIF(iso)4G, and the insertion of the E309K mutation in eIF(iso)4G confers resistance in plants, diminishing the interaction with VPg [42]. In this study, we demonstrated a direct interaction between RBSDV P8 and the N-terminal domain of rice eIF4G both in yeast and in planta using yeast two-hybrid and luciferase complementary imaging (LCI) assays (Figure 5). Deletion of the 79-site proline mutation in eIF4G generated via CRISPR/Cas9, which confers partial resistance to RBSDV in plants, did not diminish the interaction with P8 (Figure 5). These results suggest that RBSDV P8 may recruit rice eIF4G to facilitate virus infection, and the conferred partial resistance to viruses of *eif4g* mutant plants may be due to the decreased transcriptional level of *eIF4G* in mutant plants (Figure 1E). Additional experiments are needed to reveal the precise relationship between rice *eIF4G* and RBSDV proteins and whether the *eIF4G-P8* interaction functions as a convergence node of the viral cycle.

## Data Availability

Not applicable.

## Supplementary Materials

The following are available online at https://www.mdpi.com/article/10.3390/v13102100/s1, Figure S1. Evaluation of rice stripe virus (RSV) resistance of rice *eif4g* mutant lines, Table S1: Primers sequences used for cloning of transgenes and testing the viral infection.
