# Supplementary material for: Genome Editing of Rice eIF4G Loci Confers Partial Resistance to Rice Black-Streaked Dwarf Virus
Source: Viruses. 2021 Oct 18;13(10):2100. doi: 10.3390/v13102100 (PMC8539751; doi:10.3390/v13102100)
Supplement: Supplementary file 1 [file viruses-13-02100-s001.zip › viruses-1381796-supplementary.pdf]

viruses

Table S1. Primers sequences used for cloning of transgenes and testing the viral infection.

Gene	Primers	Product Size (bp)	tests
eIF4G	5'-GGCGAGGGATTTATGTCCCAGCG-3'<br>5'- CGCTGGGACATAAATCCCTCAAA-3'	oligo	CRISPR clone
eIF4G	5'-CAAGGTTCGCTCCCCTTTTTTTCCC-3'<br>5'-GGAGCTGAGCTTGTCCTGGCAGGGA-3'	1620	PCR
eIF4G-N	5'- GGGGACAAGTTTGTACAAAAAAGCAGG CTTCTATGTCCCAGCGAGGGGACAGG -3'<br>5'- GGGGACCACTTTGTACAAGAAAGCTGGGT CTTATTGATGATGGTGCTGAAT -3'	1053	Gateway clone
P8	5'-GGGGACAAGTTTGTACAAAAAAGCAGG CTTCTATGACTGGCACCCATGACGA-3'<br>5'-GGGGACCACTTTGTACAAGAAAGCTGGGT CTACAATAATCGAGGAAGCTT-3'	1773	Gateway clone
P9-1	5'-AAGAGCGGAGAACGTTTGGA-3'<br>5'-CGAAGATGCCGTCATCGAGT-3'	150	qRT-PCR
eIF4G	5'-CCTGGGATGCCCATGTCAAT-3'<br>5'-TGCATCATTGTTGGCGGTTG-3'	209	qRT-PCR
SP	5'-TTGTCACTCATTCTTATCACACCTG-3'<br>5'-TTCTTCCACACTTTCTCATACTCTT-3'	237	qRT-PCR
UBQ10	5'-TGGTCAGTAATCAGCCAGTTTGG-3'<br>5'-GCACCACAAATACTTGACGAACAG-3'	65	qRT-PCR
Cas9	5'-AAAGACCGAGGTGCAGACAG-3'<br>5'-CGATCCGTGTCTCGTACAGG-3'	794	PCR

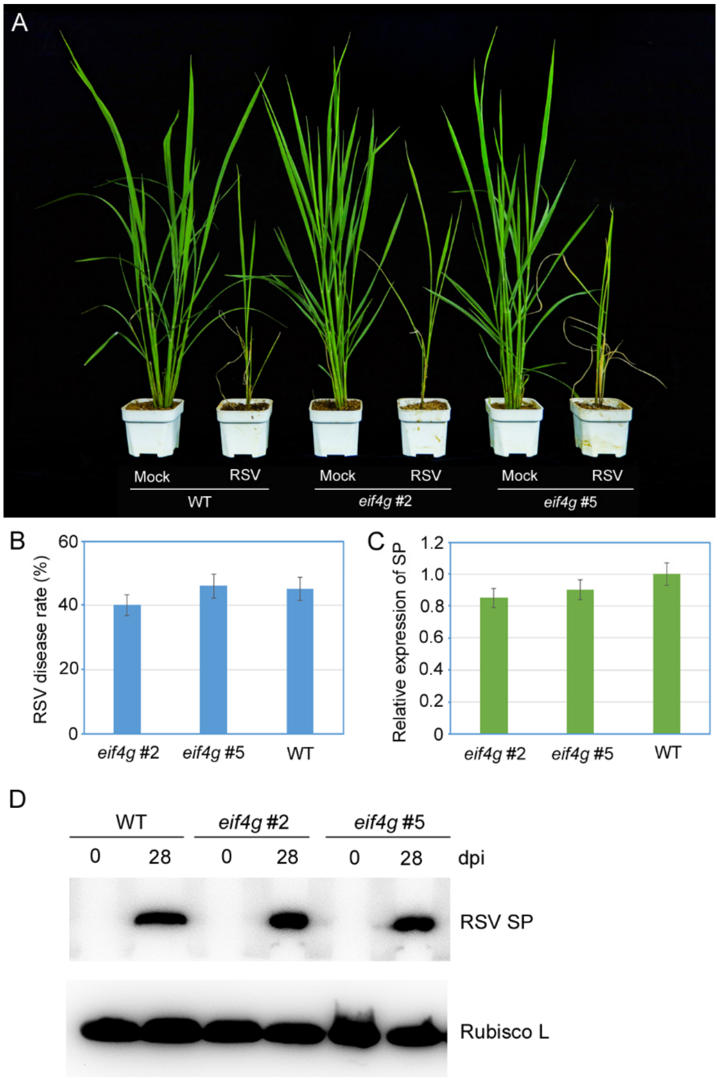

Figure S1. Evaluation of rice stripe virus (RSV) resistance of rice *eif4g* mutant lines. (A) Disease symptoms of mock-inoculated and RSV-infected *eif4g* mutant lines (*eif4g* #2, *eif4g* #5) and wild-type (WT, Nipponbare) rice plants. Photographs were taken at 28 days postinoculation (dpi). (B) Incidence of RSV disease rate in *eif4g* mutant lines (*eif4g* #2, *eif4g* #5) and wild-type (WT, Nipponbare) rice plants at 28 dpi. (C) qRT–PCR analysis of RSV *SP* mRNA transcription levels in *eif4g* mutant lines (*eif4g* #2, *eif4g* #5) and WT rice plants at 28 dpi. Signal intensities for each transcript were normalized to the signal intensity for UBQ. (D) Western blot analysis of RSV-encoded SP protein accumulation in virus-infected *eif4g* mutant lines (*eif4g* #2, *eif4g* #5) and wild-type (WT, Nipponbare) rice plants using a SP-specific antibody. The rubisco large subunit level served as a loading control. Rice plants were all collected at 28 dpi. All data are means ± SD (n = 3).
